# Prior *Toxoplasma Gondii* Infection Ameliorates Liver Fibrosis Induced by *Schistosoma Japonicum* through Inhibiting Th2 Response and Improving Balance of Intestinal Flora in Mice

**DOI:** 10.3390/ijms21082711

**Published:** 2020-04-14

**Authors:** Fei Xu, Ruitang Cheng, Sunhan Miao, Yuwei Zhu, Ze Sun, Liying Qiu, Junqi Yang, Yonghua Zhou

**Affiliations:** 1 Department of Basic Medicine, Wuxi Medical School, Jiangnan University, Wuxi 214122, Jiangsu, China; feixuhgd@163.com (F.X.); crt318200@163.com (R.C.); 1282180210@stu.jiangnan.edu.cn (S.M.); wopianbugaosuni1@163.com (Y.Z.); qiulydoc@jiangnan.edu.cn (L.Q.); 2 Key Laboratory of National Health Commission on Parasitic Disease Control and Prevention, Jiangsu Provincial Key Laboratory on Parasite and Vector Control, Jiangsu Institute of Parasitic Diseases and Public Health Research Center of Jiangnan University, Wuxi 214064, Jiangsu, China; Junqi.Yang@cchmc.org; 3 College of Mechanical Engineering, Donghua University, Shanghai 201620, China; sun_3717@163.com; 4 Division of Experimental Hematology and Cancer Biology, CinCinnati Children’s Hospital Medical Center, CinCinnati, OH 45229, USA

**Keywords:** *Toxoplasma gondii*, *Schistosoma japonicum*, parasitic co-infection, liver fibrosis, T helper cell type 2, gut microbiome, gut-liver axis

## Abstract

Schistosomiasis is an immunopathogenic disease in which a T helper (Th) cell type 2-like response plays vital roles. Hepatic fibrosis is its main pathologic manifestations, which is the leading cause of hepatic cirrhosis. Co-infections of *Schistosoma japonicum* (Sj) with other pathogens are frequently encountered but are easily ignored in clinical studies, and effective therapeutic interventions are lacking. In this study, we explored the effect of *Toxoplasma gondii* (Tg) prior infection on Th1/Th2 response, community shifts in gut microbiome (GM), and the pathogenesis of schistosomiasis in murine hosts. Mice were prior infected with Tg before Sj infection. The effects of co-infection on Th1/Th2 response and hepatic fibrosis were analyzed. Furthermore, we investigated this issue by sequencing 16S rRNA from fecal specimens to define the GM profiles during co-infection. Tg prior infection markedly reduced the granuloma size and collagen deposit in livers against Sj infection. Prior infection promoted a shift toward Th1 immune response instead of Th2. Furthermore, Tg infection promoted the expansion of preponderant flora and Clostridiaceae was identified as a feature marker in the GM of the co-infection group. Redundancy analysis (RDA)/canonical correspondence analysis (CCA) results showed that liver fibrosis, Th1/Th2 cytokines were significantly correlated (*P* < 0.05) with the GM compositions. Tg infection inhibits hepatic fibrosis by downregulating Th2 immune response against Sj infection, and further promotes the GM shifts through “gut–liver axis” in the murine hosts. Our study may provide insights into potential anti-fibrosis strategies in co-infection individuals.

## 1. Introduction

Schistosomias is an immunopathogenic disorder due to the deposition of trematodes ovum in the small portal venules, finally causing periportal and liver cirrhosis [1,2]. It remains a severe public health problem in many developing countries. The formation of hepatic egg granulomas and fibrosis are the primary causes of mortality in human with schistosomiasis [3]. Recent studies have shown that CD4^+^ T cell response plays a key role in the development of liver fibrosis. The polarization of T helper (Th) cell type 1/Th2 response and their immunoregulation are closely related to the occurrence and development of schistosomiasis-induced hepatic fibrosis [4,5,6,7,8,9,10]. 

*Toxoplasma gondii* (*T. gondii*) is an obligatory intracellular parasite that is capable of infecting all homoeothermic animals including human beings, which causes lethality in immunocompromised individuals. Chronic infection of *T. gondii*, usually presents an asymptomatic state in clinical. But it may be related to a wide range of abnormality, including encephalitis, Parkinson’s disease, schizophrenia, obsessive-compulsive disorder and Alzheimer’s disease [11,12,13,14,15]. *T. gondii* secretes effector molecules from secretory organelles into the host cytosol that modulate host signaling pathways. Among them, rhoptry kinase 16 (ROP16) and dense granule protein 15 (GRA15) have significant effects on the cell transcription of hosts. Studies have shown that GRA15-transfected macrophages can induce M1 cell activation in mouse infected with type II Toxoplasma. However, macrophages of hosts infected with type I or III Toxoplasma are polarized towards an M2 state [16,17,18,19]. Although no studies have directly implicated the role of *T. gondii* infection in liver diseases, approximately 30% of individuals with chronic liver diseases were tested as *T. gondii* B1 serum positive compared to 6% in control samples. Furthermore, these *T. gondii* seropositive patients are accompanied with elevated circulating aspartate aminotransferase (AST) and alanine aminotransferase (ALT) levels [13]. These researches indicate that *T. gondii* infection may actively participate in granuloma formation and fibrosis.

The gut microbiota (GM) exist in a specific symbiotic order within the human body [20,21]. Generally, it is well known that GM plays critical roles in pathological and physiological conditions relevant to human health, like taking part in vitamin B synthesis, digestion, immune system modulation, and promotion of nerve function [22,23,24]. A growing body of literature is showing that GM has a relevant impact on the pathogenesis of hepatic, respiratory, endocrine, and many other disorders, and refers to it as a “virtual metabolic organ”. GM form axes with a number of extraintestinal organs, but among them the gut–liver axis gains increasing attention in recent years [25]. Due to a close anatomical and bidirectional interaction of the liver and gastrointestinal tract, the gut-liver interaction is stabilized primarily through the portal circulation [26]. Because of the presence of the hepatic axis, liver lesions are often closely associated with intestinal lesions and microecological composition. The population shifts in GM play a regulatory role in pathogenesis of many chronic liver diseases like chronic hepatitis B/C, non-alcoholic fatty liver, liver cirrhosis [27] and hepatocellular carcinoma [28]. Qin at el. found that Bacteroidetes and Firmicutes levels reduced, while Veillonella spp. and Streptococcus spp. levels increased in GM of cirrhotic patients, in comparison with healthy population [29]. Bajaj et al., showed that a significant improvement of GM symbiosis and diversity was found in a patient with severe cirrhosis after liver transplantation [30]. Since the gut–liver axis has a critical impact on the pathogenesis of chronic liver diseases, it emerges as a clinical focus of current research. The correlation between hepatic fibrosis and the key functional flora in intestinal microecology remains unclear, needless to say under parasite infection conditions.

Although it is often neglected in clinical, superimposed infection of different parasites is a common phenomenon in patients. Infection of one type of parasite can trigger immune response in the host, which may affect subsequent infection of another parasite and finally change the pathogenic outcome. Hosts are more susceptible to chronic infections than to acute ones, as most primary infections are usually asymptomatic or occasionally with mild symptoms in healthy individuals. In spite of the increasing interests in understanding the complex superposition effect of co-infection, studies investigating the impact of *S. japonicum* and *T. gondii* co-infection are scant. 

In the current study, we first evaluated the effect of chronic *T. gondii* prior infection on hepatic fibrosis induced by *S. japonicum* in a murine infection model. We then explored the underlying cellular mechanisms through which *T. gondii* affects the pathogenesis of schistosomiasis. We found that *T. gondii* prior infection is able to inhibit the granuloma formation and subsequent hepatic fibrosis. This inhibition is related to the suppression of Th2 as well as the shift to Th1 response. Furthermore, hepatic fibrosis and Th1/Th2 immune response all significantly influenced the bacterial community structures (redundancy analysis/canonical correspondence analysis results). Clostridiaceae was identified as featured preponderant bacteria in the GM of the co-infection group. The current study might serve as a potential therapeutic strategy for hepatic fibrosis due to *T. gondii* and schistosome infection.

## 2. Results

### 2.1. Co-Infection of Toxoplasma gondii and Schistosoma japonicum Leads to Decreased Liver Function 

**Figure 1:**
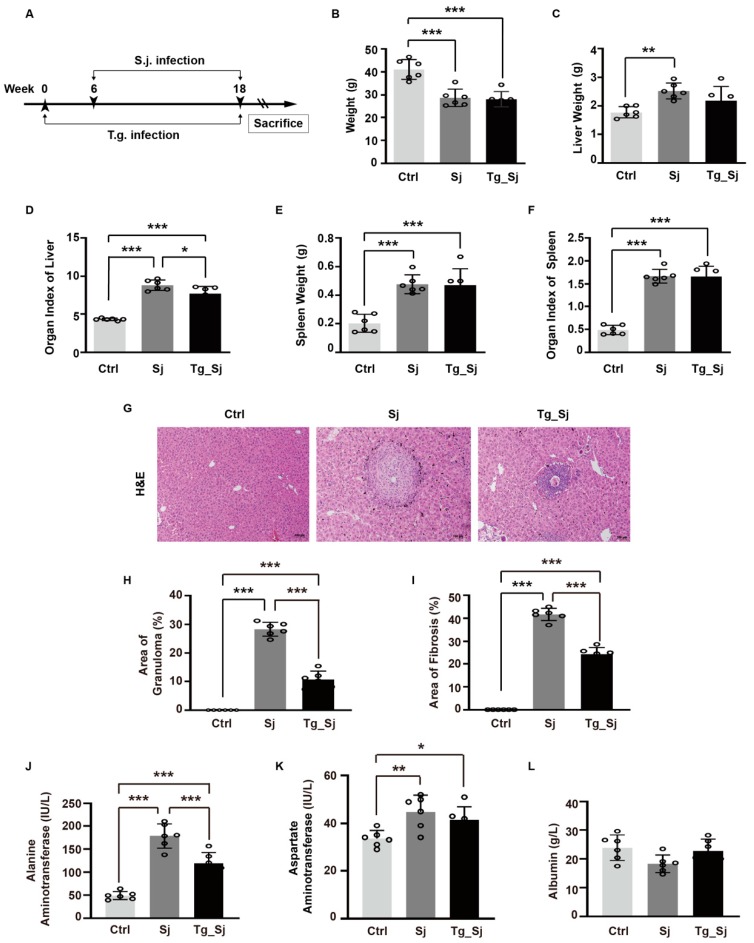
Co-infection of *Toxoplasma gondii* (*T. gondii*) and *Schistosoma japonicum* (*S. japonicum*) lead to decreased liver function. (**A**) Infection scheme of the mice model. Female ICR mice were infected with *T. gondii* six weeks before *S. japonicum* infection. Mice were sacrificed 18 weeks after the primary *T. gondii* (Tg) injection. (**B**) Body weight (BW) and organ weight coefficients of mice. (**C**,**E**) Liver and spleen weight of mice in control (Ctrl), *S. japonicum* (Sj)-infected and co-infected (Tg_Sj) groups. (**D**,**F**) Liver and spleen index (organ weight/BW) of mice. (**G**) Representative hematoxylin and eosin (H&E) staining datas were shown in liver sections (scale bar, 100 μm). (**H**) The sizes of granulomas and (**I**) quantification of fibrotic area areas around a single egg were measured. The percentage of Sj and Tg_Sj group to the control group was calculated. (**J**,**K**,**L**) Albumin and serum aspartate aminotransferase, alanine aminotransferase levels were analyzed by automatic biochemical analyzer. Results are representative of at least three independent experiments (B-L, *n* = 6, one-way ANOVA). * *p* < 0.05, ** *p* < 0.01, *** *p* < 0.001.

A mice model was constructed by prior infecting with *Toxoplasma gondii* (*T. gondii*) before *Schistosoma japonicum* (S. *japonicum*) infection (Figure 1A), which could be considered as a mimic pathological model in clinical. We first found that the body weight of both *S. japonicum* mono-infected and co-infected mice decreased on the day of sacrifice (Figure 1B). A significant difference of liver weight and organ index were observed in the mono-infected group when compared with control group, among which liver index was attenuated in co-infection group (Figure 1C,D). Although spleen weight and organ index were significantly increased in mono-infection mice, none of these two indexes changed significantly with *T. gondii* prior infection, when compared with *S. japonicum* infection group (Figure 1E,F). Mice were sacrificed 18 weeks after the primary injection, we observed obvious hepatic granulomas and fibrosis in infected groups. The size of the granulomas around a single egg was measured in liver sections with hematoxylin and eosin (H&E) staining (Figure 1G). *T. gondii* prior infected mice showed much smaller granulomas than *S. japonicum* mice (Figure 1H). The fibrotic area around the granulomas was significantly reduced in co-infected group (Figure 1I). Serum levels of aspartate aminotransferase (AST), alanine aminotransferase (ALT) and albumin (ALB) were measured to reflect the basic function of liver. Decreased levels of ALT and AST were observed in co-infected mice, when compared to *S. japonicum* mono-infected group (Figure 1J,K). Slightly decreased levels of ALB were observed in *S. japonicum* group, which was reversed by *T. gondii* prior infection, although showed no significance after analysis (Figure 1L). Taken together, histopathological and biochemical analysis supported an antagonism effect of prior *T. gondii* infection against *S. japonicum* to liver function. 

### 2.2. Prior Infection with Toxoplasma gondii Attenuates Hepatic Fibrosis in Mice Infected with Schistosoma japonicum 

**Figure 2:**
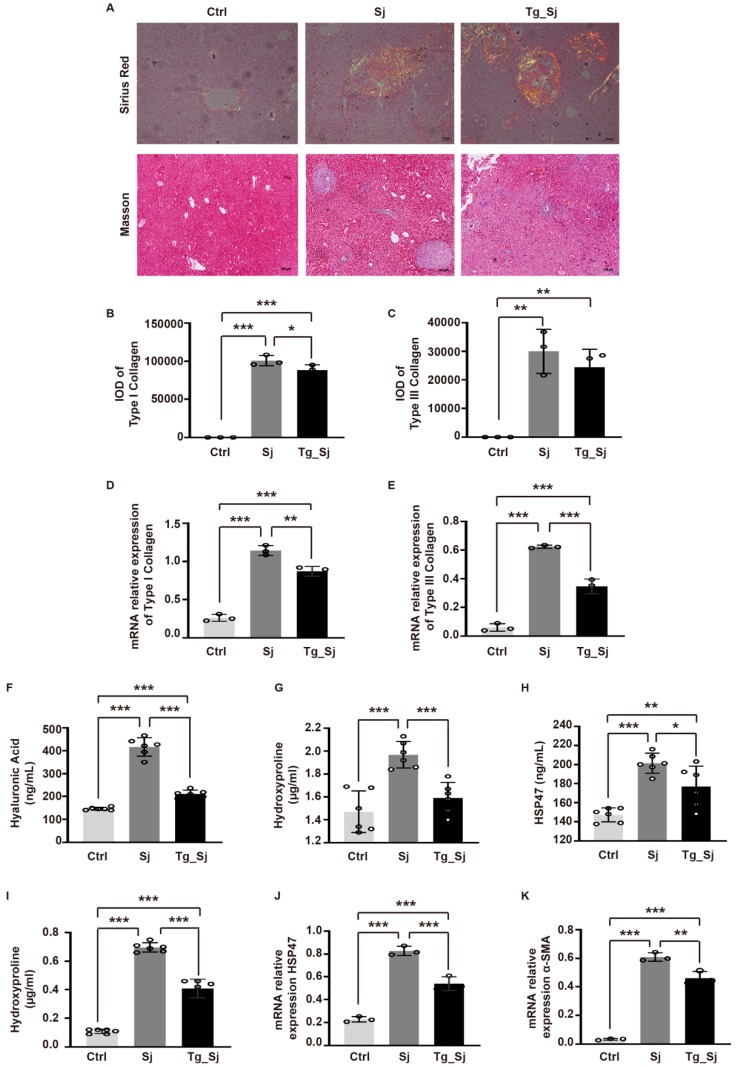
Co-infection of *T. gondii* and *S. japonicum* suppresses hepatic granuloma formation and fibrosis in mice. (**A**) Upper panel: Sirius red staining of collagen type I (orange or red) and collagen type III (green); Lower Panel: Masson trichrome staining of collagens (blue), images were taken by polarized light microscope, scale bar 100 μm. (**B**,**C**) Quantification of collagen type I and type III by Image pro plus 6.0. (**D**,**E**,**J**,**K**) The mRNA levels of collagen type I, type III, heat shock protein 47 (HSP47) andα-smooth muscle actin (SMA) in liver tissues were determined using real-time PCR. Data are normalized to GAPDH and expressed as arbitrary units. (**F**,**H**) The concentrations of serum hyaluronic acid (HA), HSP47 were determined by ELISA. Hydroxyproline (HYP) in serum (**G**) and in liver tissues (**I**) was detected by alkali pyrolysis and analyzed with semi-automatic biochemical analyzer (colorimetric wavelength: 550 nm). Results are representative of at least three independent experiments (**B**–**E**,**J**,**K**, *n* = 3, F-I, *n* = 6, one-way ANOVA). * *p* < 0.05, ** *p* < 0.01, *** *p* < 0.001.

Hence, we further confirmed the potential effects of *T. gondii* (Tg) in the hepatic fibrosis pathogenesis of *S. japonicum* (Sj) infection. Hyaluronic acid (HA) level is positively correlated with the degree of liver fibrosis [31]. Our data revealed that prior infection with *T. gondii* alleviated the HA level when compared with *S. japonicum* mono-infection group (Figure 2F). Heat shock protein 47 (HSP47) is the molecular chaperone required for normal, synthesis and secretion of procollagen [32]. Hepatic hydroxyproline (HYP) is a signature amino acid for fibrillar collagens [33]. Consistent with the above findings, we found both serum and mRNA levels in liver tissues of HYP and HSP47 were increased in mono-infected mice as compared with control group, but could be significantly reversed by co-infection (Figure 2H,J). The activation of hematopoietic stem cell (HSC) located within the perisinusoidal space is an important process of liver fibrosis. α-smooth muscle actin (SMA) is a sign of HSC activation [34] and the mRNA level of α-SMA in liver tissues sharply increased after *S. japonicum* infection, but could be significantly attenuated by *T. gondii* prior infection (Figure 2K). Type I and III collagen are the main extracellular basal components of liver fibrosis. The inhibition of collagen formation is the key strategy to control the process of liver fibrosis. Here, the expression of type I and III collagen in the *S. japonicum* group was significantly increased, and the morphology showed cord-like extension to the hepatic sinus to separate hepatic lobules. The co-infection could reduce the contents of collagen fibers I and III (Figure 2A, upper panel), although that of collagen fibers III showed no significance after analysis (Figure 2B,C). The fibrotic areas around the granulomas were significantly reduced in co-infection groups as confirmed by Masson trichrome staining (Figure 2A, lower panel). To further verify the morphology results, we detected the mRNA expressions levels of collagen type I and III. The mRNA levels in co-infection group were significantly lower than those in *S. japonicum* mono-infected mice, but still higher than the control group (Figure 2D,E). Collectively, our data suggest that *T. gondii* prior infection can suppress hepatic granuloma formation and subsequent fibrosis induced by *S. japonicum* infection.

### 2.3. Co-Infection Inhibits Schistosoma japonicum Mediated Th2 Differentiation and Lead to Th1 Response 

**Figure 3:**
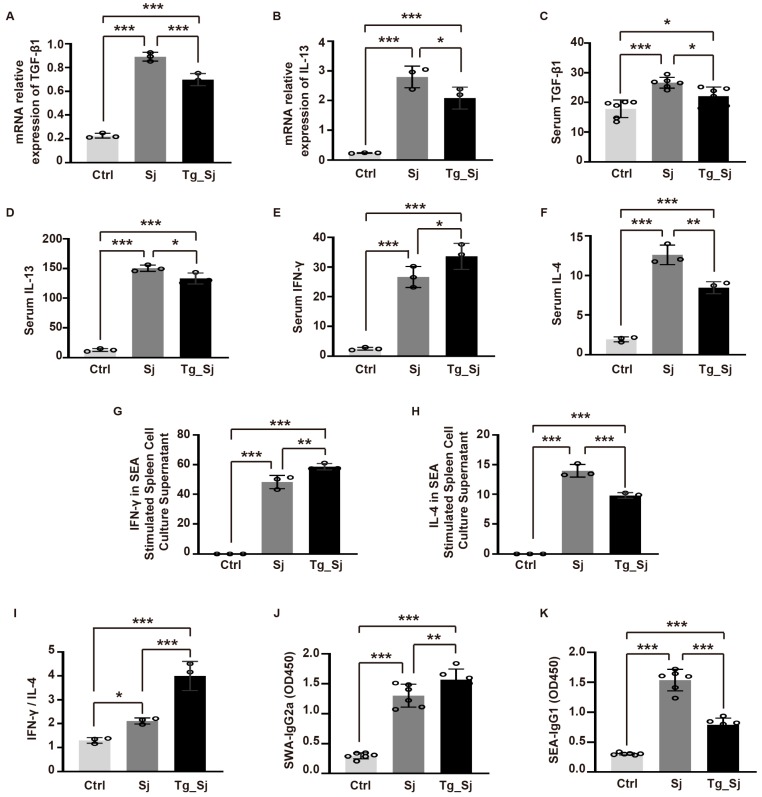
Co-infection inhibits *S. japonicum* mediated Th2 immune response. (**A**,**B**) The mRNA levels of TGF-β1, IL-13 in liver tissues were determined using real-time PCR. Data are normalized to GAPDH and expressed as arbitrary units. (**C**,**D**,**E**,**F**) The serum levels of TGF-β1, IL-13, IFN-γ and IL-4 were determined by ELISA. (**G**,**H**) IFN-γ and IL-4 levels in cultured supernatants from SEA-stimulated spleen cells were determined by ELISA. (**I**) The ratio between IFN-γ and IL-4 levels. (**J**) SWA-IgG2a and (**K**) SEA-IgG1 levels was detected by ELISA. Results are representative of at least three independent experiments (**A**,**B**,**D**–**I**, *n* = 3, **C**,**J**,**K**, *n* = 6, one-way ANOVA). * *p* < 0.05, ** *p* < 0.01, *** *p* < 0.001.

Soluble egg antigen (SEA) secreted by schistosoma larva can activate the hosts’ immune response and promotes the secretion of Interleukin (IL)-13 [35], which further activates macrophages to secrete transforming growth factor-β (TGF)-β1. TGF-β1 can regulate the size of granulomas and promote collagen synthesis. To assess the effect of prior *T. gondii* infection on fibrosis relevant immune response of the hosts, the mRNA levels of TGF-β1 and IL-13 in livers were investigated with real-time PCR (Figure 3A,B) and the levels in serum supernatant were detected by ELISA (Figure 3C,D). Compared with the *S. japonicum* mono-infected group, the levels of TGF-β1 and IL-13 in co-infected group were decreased. 

CD4+ T cells play an important role in the pathogenesis of schistosomiasis, which differentiate into T helper (Th) cell type 1/Th2 subsets after being stimulated by schistosoma antigen. Th1 cells mainly secrete cytokines such as IL-2, Interferon (IFN)-γ, tumor necrosis factor (TNF)-α to mediate hosts’ immune response. While, Th2 cells secrete IL-4, IL -5, IL -6, IL -10 and IL -13. Normally, the ratio of Th1/Th2 is in dynamic balance, which may be disrupted by specific antigen stimulation and results in a drift of Th1/Th2 cell subsets. IFN-γ can promote the differentiation of Th0 into Th1 cells, while IL-4 is necessary for the differentiation of Th2 cells [36,37]. Compared with control group *S. japonicum* infection dramatically increased the serum IFN-γ levels of the hosts. Meanwhile, *T. gondii* co-infection has a synergistic effect on IFN-γ secretion (Figure 3E). On the contrary, prior infection with *T. gondii* attenuated IL-4 secretion when compare with *S. japonicum* group (Figure 3F). It was noteworthy that, the ratio of IFN-γ over IL-4 was sharply enhanced in co-infection group (Figure 3I).

It has been reported that in the course of schistosomiasis infection, soluble worm antigen (SWA) of *S. japonicum* can induce the transformation of macrophages into M1-type, and subsequently enhance the Th1 cell response. After eggs are produced, the soluble egg antigen (SEA) upregulated Th2 cells. Thus, the levels of SWA-IgG2a and SEA-IgG1 could reflect the responses of Th1 and Th2 cells respectively [38]. In order to further verify the response of Th1 and Th2 cells, the expression levels of anti-SWA-IgG2a and anti-SEA-IgG1 in serum of each group were detected. Compared with the control group, the expression of anti-SWA-IgG2a in the mono-infected and co-infected groups increased significantly, indicating co-infection had a synergistic effect on SWA-IgG2a secretion (Figure 3J). It was not surprising that, *T. gondii* exerted an antagonism effect on anti-SEA-IgG1 expression levels, with a sharply decreased OD450 in co-infected group (Figure 3K). SEA collected in serum was further used to stimulate splenic lymphocytes in vitro. The results showed that a large release of IFN-γ was observed in the supernatant of splenic cell culture of co-infection group, which was slightly higher than the mono-infection group (Figure 3G). On the contrary, the secretion of Th2 cytokine IL-4 was significantly inhibited by co-infection of *T. gondii* (Figure 3H). Above results indicated previous infection of *T. gondii* significantly inhibits the activation of T cells, prior infection of *T. gondii* influenced the polarization of Th1/Th2 response, which inhibited the drift of immune response from Th1 to Th2. 

### 2.4. Structure Succession Analysis of Gut Microbiota Community Composition 

Preclinical studies hint that intestinal flora may play key roles in the progression of various liver-related disease. Chikano et al. showed that intestinal flora and mucosal inflammation induce fatty liver in an IFN-γ dependent manner in BALB/c-wt mice [39]. Another study showed that bacterial translocation is linked to the over-expression of IFN-γ, IL-4 in a cholestasis model [40]. We further investigated this issue by sequencing 16S rRNA amplicon DNA from fecal specimens to define the GM community profiles during co-infection. A total of 12 samples (3 samples in each group of Ctrl, Tg, Sj, Tg_Sj) from ICR mice were collected for 16S rRNA sequencing. Pre-processing, including de-noising and filtering of the sequence datasets, resulted in analysis of a total 418,298 sequences for 12 baseline samples with an average of 35,559 (Standard deviation: 4323) sequences per sample. The number varied from a maximum of 43,777 sequences obtained in *T. gondii* group (sample Tg_3), to a minimum of 30,200 sequences obtained in *S. japonicum* infected group (sample Sj_2).

**Figure 4:**
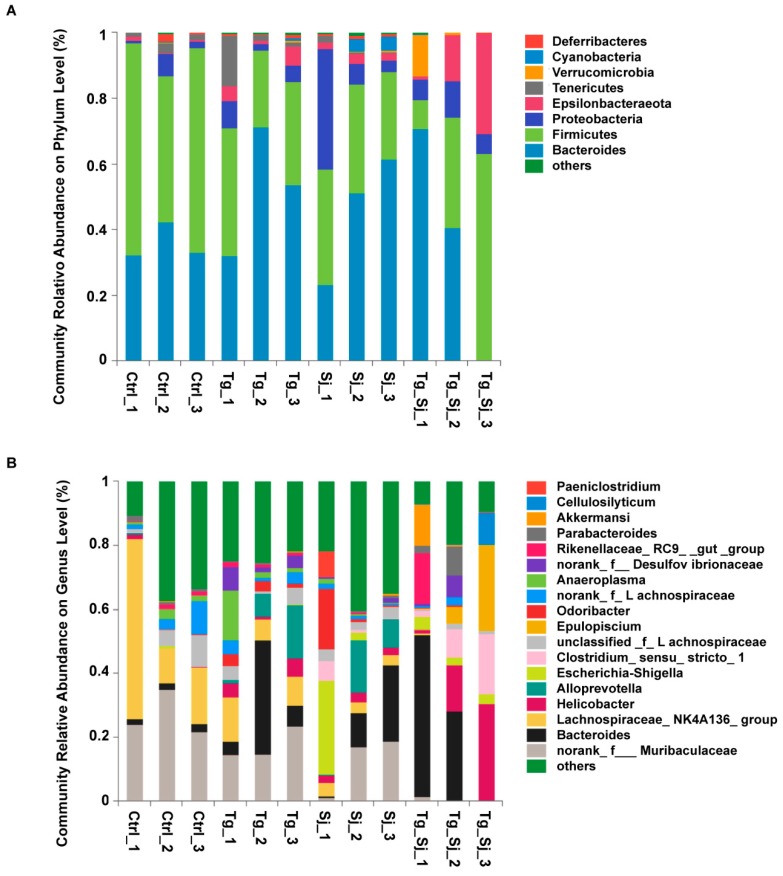
Variation of bacterial community composition of the intestinal microbiota at two taxonomic levels. This bar chart showed the relative abundance of the most variable bacteria at (**A**) phylum and (**B**) genus levels present in gut. Three independent samples were included in control (Ctrl), *T. gondii* (Tg), *S. japonicum* (Sj)-infected and co-infected (Tg_Sj) groups. Low abundance and unclassified bacteria were grouped in “Others”.

Across all groups, the phyla Bacteroidetes and Firmicutes were the most abundant (>60%), except in one sample from the co-infection group (sample Tg_S_3), which is dominated by Firmicutes (Figure 4A). For the Bacteroidetes, the range spanned from 0.07% (sample Tg_Sj_3) to 71.3% (sample Tg_2). The minimum and maximum abundance of Firmicutes were 8.8% (sample Tg_Sj_1) and 64.5% (sample NC_1), respectively. The ratio of Firmicutes over Bacteroidetes remained above 1.0 across all but six samples (samples Ctrl_1, Ctrl_2, Ctrl_3, Sj_1, Tg_Sj_3 and Tg_1), which were found mainly in non-infected animals. The Proteobacteria phylum was the third most abundant taxon, with a median of 8.52% across all samples. While the relative abundance of this microbiome was low in the normal control group, with a median of 2.45% (minimum of 2.16%; maximum of 7.07%). The median value in animals infected with *S. japonicum* was 9.54% (minimum of 5.94%; maximum of 39.1%), while in co-infection group was 26.0% (minimum of 7.49%; maximum of 36.5%). The ratio of Firmicutes over Proteobacteria was above 1.0 in all except for one sample from the *S. japonicum* infected groups (samples Sj_1). The ratio was high in the normal control group, with a median of 25.5 (minimum of 6.24; maximum of 29.93) but was low in animals infected with *T. gondii* and *S. japonicum*, with a median of 1.30 (minimum of 1.17; maximum of 1.73). Moreover, the ratio of Bacteroidetes over Proteobacteria was above 1.0 in all but two samples, from the *S. japonicum* and co-infection group (samples Sj_1 and Tg_Sj_3). A final observation at the phylum level was the presence of Tenericutes, Verrucomicrobia, Deferribacteres, and Cyanobacteria with a median of 1.04%, 0.04%, 0.29%, and 0.07% respectively. Among them, Deferribacteres (median value: 0.00% in Ctrl, 0.04% in Sj, 0.55% in co-infection group) was a dominant bacterial community functions in the progression of liver fibrosis.

At the genus level, the abundance of Lachnospiraceae_NK4A136, a subgroup of the phylum Firmicutes, reached a maximum of 56.6% in the control group (minimum of 10.1%) (Figure 4B). Whereas in co-infected group, the abundance of the same genus was as low as 0.047% of the overall composition (Median: 0.076%). That is significantly lower than *S. japonicum* mono-infected group (*student’s t-test*, *P* = 0.0051). The same tendency was found in Bacteroidales_S24-7_group_norank (median value: 24.4% in Ctrl, 17.1% in Sj, 0.052% in co-infection group), a subgroup of the phylum Bacteroidetes. Meanwhile, the abundance of 5 taxon increased in co-infection group compared with control group, they were Helicobacter (median value: 0.29% in Ctrl, 2.31% in Sj, 14.7% in co-infection group), Bacteroides (median value: 2.27% in Ctrl, 11.0% in Sj, 26.9% in co-infection group), Escherichia-Shigella (median value: 0% in Ctrl, 2.74% in Sj, 2.93% in co-infection group), Epulopiscium (median value: 0.00% in Ctrl, 0.00% in Sj, 5.43% in co-infection group) and Clostridium_sensu_stricto_1 (median value: 0.02% in Ctrl, 0.80% in Sj, 9.17% in co-infection group). The abundance of members in subgroups of Firmicutes was highly heterogeneous among the different groups *T. gondii*.

### 2.5. Toxoplasma gondii Prior Infection Reduces the Gut Microbial Alpha Diversity

**Figure 5:**
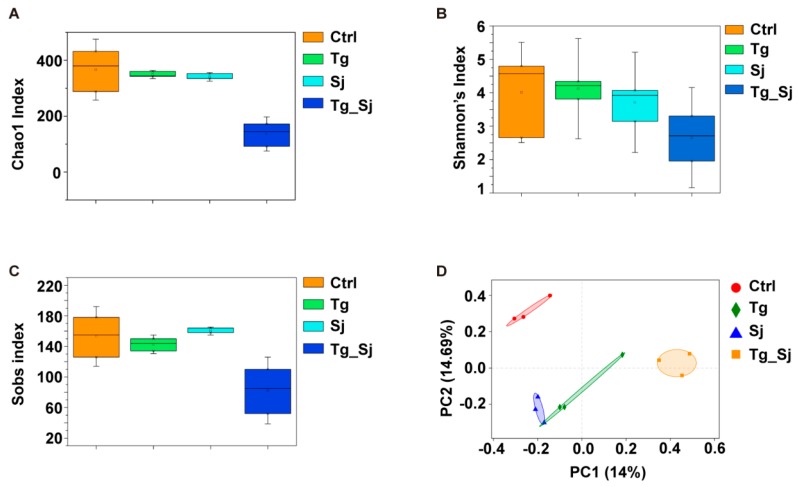
Diversity comparison of bacterial community in different groups. The gut microbial profiles of mice ordinated by alpha diversity analysis based on the (**A**) Chao1 index, (**B**) Shannon’s index, (**C**) observed species (Sobs) index among groups control (Ctrl), *T. gondii* (Tg), *S. japonicum* (Sj)-infected and co-infected (Tg_Sj) groups. (**D**) Beta diversity analysis based on bray curtis in different groups.

Alpha diversity was calculated to reflect the influence of parasites infection on the structure of gut microbiota. For Chao1 and sobs indexes analysis (Figure 5A,C), co-infected samples had a significantly lower value than control (*P* = 0.0087 and (*P* = 0.0351 respectively) and *S. japonicum* mono-infected group (*P* = 0.0011 and *P* = 0.0101 respectively). Shannon index was decreased in the microbiota of Tg_Sj group when compared with control and *S. japonicum* mono-infected group, although these changes were not statistically significant (*P* = 0.1594 and *P* = 0.0951) (Figure 5B). To measure the level of similarity between gut microbial communities. GM were clustered by Principal Coordinates Analysis (PCoA), which clearly separated samples of co-infected mice from all other samples. On the PCoA plot, each unique symbol represents the GM of one certain group. It is noteworthy that the flora from control group and co-infection group were significantly distinct between each other (Figure 5D). The between-group distances were significantly higher than the within-group distances (ANOSIM R value = 0.7, *P* = 0.001) for each group. Above data suggested that the structures of gut microbial community between co-infected group and the other groups were significantly different.

**Figure 6:**
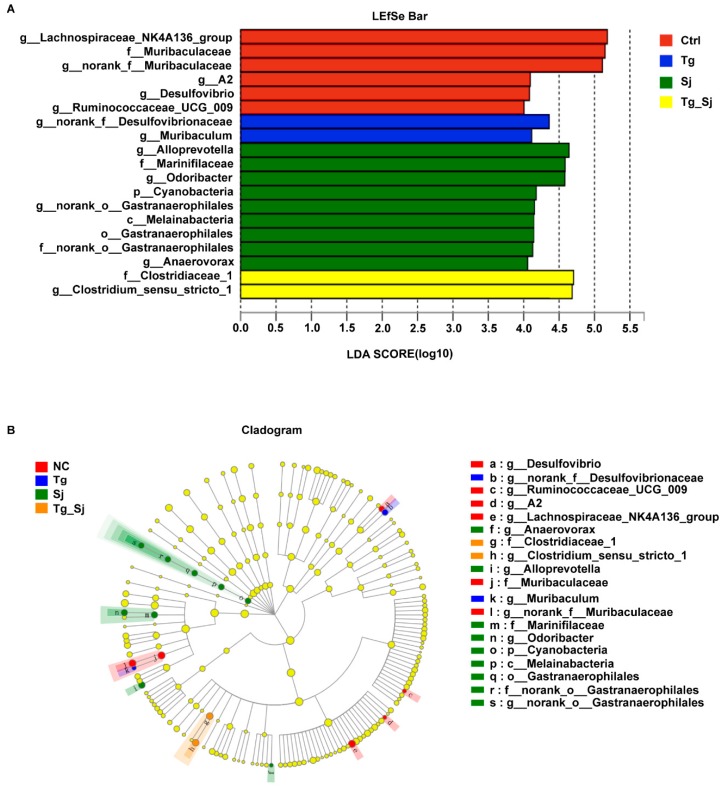
The most differentially abundant taxa identified by LEfSe in mice. (**A**) LDA score distribution among control (Ctrl), *T. gondii* (Tg), *S. japonicum* (Sj)-infected and co-infected (Tg_Sj) groups. (**B**) Cladogram of the most differentially abundant taxa in different groups. Only taxa meeting a histogram of linear discriminant analysis (LDA) significance threshold >4 are shown in the figures.

To identify changes in the fecal microbiota community, LEfSe was used to compare the microbial species present in the guts of each group at different taxonomic levels (Figure 6A). There were five genera that showed the highest relative abundance in the control group: Muribaculaceae originating from Muribaculaceae, as well as Desulfovibrio, Ruminococcaceae_UCG_009, A2 and Lachnospiraceae_NK4A136_group. Following *S. japonicum* infection, there were a key family and three key genera in groups. Specifically, Marinifilaceae originating from Odoribacter, as well as Alloprevotella were most abundant in the *S. japonicum* mono-infection group. A key family, Clostridiaceae, belonging to the Clostridium_sensu_stricto_1 at genus level, was most abundant in the co-infected group. This was a biomarker with a significant difference between the co-infected group and the other three groups.

### 2.6. Correlations of Bacterial Community with Liver Fibrosis and Th1/Th2 Response

**Figure 7:**
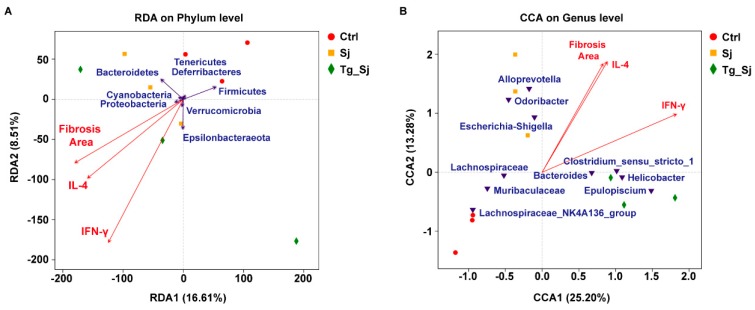
Redundancy analysis (RDA) and canonical correspondence analysis (CCA) based on microbial community and environmental factors. (**A**) RDA for bacterial communities at phylum level. (**B**) CCA for bacterial communities at genus level. Red arrows represented environmental variables. Polygon symbols (in red, yellow, green) and associated name represented samples. Inverted triangle (in blue) indicated the environmental factors of significant influence on bacterial communities.

To explore the influence of potential factors, the variations of bacterial community, redundancy analysis (RDA)/canonical correspondence analysis (CCA) analysis was performed to reveal the correlations between the measured liver fibrosis, Th1/Th2 cytokines and the dominant microbial communities at the phylum or genus level (Figure 7). As shown in Figure 7A, RDA showed that the cumulative contribution rate of factors to the structure of the flora was 16.61% and 8.51%, respectively. At the phylum level, the RDA result of samples demonstrated that Epsilonbacteraeota, Proteobacteria, and Verrucomicrobia were positively related with liver fibrosis, IL-4 and IFN-γ. Among them, Epsilonbacteraeota had the greatest correlation with IFN-γ. Cyanobacteria only positively correlated with liver fibrosis and IL-4, but had no correlation with IFN-γ. Bacteroidetes were positively related with liver fibrosis and IL-4, but negatively related with IFN-γ. However, Tenericutes, Deferribacteres, and Firmicutes negatively related with liver fibrosis, IL-4 and IFN-γ. The samples fermented at liver fibrosis and IL-4 were similar to each other, indicating that there were more microorganisms with similar community structures and a high correlation between these two environmental factors. The first two CCA axes explained 13.28% and 25.2% of the total variation in bacterial community. At the genus level, liver fibrosis and IL-4 which indicated Th2 response had higher correlation with Odoribacter, Alloprevotella, and Escherichia-Shigella (Figure 7B). Meanwhile, IFN-γ which indicates Th1 immune response had positively correlation with Clostridium_sensu_stricto_1, Helicobacter, Epulopiscium, and Bacteroides. On the contrary, these environmental factors negatively related with Lachnospiraceae, Muribaculaceae, and Lachnospiraceae_NK4A136_group. The above situation consistent with the community analysis previously stated that the co-infection group hold the preferred dominant bacterial group like Clostridium_sensu_stricto_1 which had higher correlation with Th1 response other than Th2 response. Above results showed that liver fibrosis (*P* = 0.008), IFN-γ (*P* = 0.004) and IL-4 (*P* = 0.003) all significantly influenced the bacterial community structures at the genus level.

## 3. Discussion

In most epidemiological investigations, the potential risks of co-infection are often overlooked. Limited studies of helminth co-infection mainly focus on acute influence on the hosts. In fact, chronic infection is more likely to represent the realistic effects of pathogen and multiple environmental factors on the host, which is different from acute infection under the concept of “one pathogen, one disease”. In this study, we first reported the mutual effects of chronic *Toxoplasma gondii* (*T. gondii*) infection on liver fibrosis induced by *Schistosoma japonicum* (*S. japonicum*) and the underlying mechanisms.

Although the statistical results of Sirius staining did not show a significant remission of collagen I and III after co-infection, but significant reduction of collagen I and III mRNA levels were observed in liver tissues. Liver fibrosis is an important pathological change of *S. japonicum* infection. Persistent fibrosis in chronic infection may cause hepatic cirrhosis with high mortality. Our results showed that the degree of hepatic fibrosis induced by *S. japonicum* could be significantly attenuated by chronic *T. gondii* infection. Although the statistical results of Sirius staining did not show a significant remission of collagen I and III after co-infection when compared with mono-infection group, a significant reduction of mRNA levels were observed in liver tissues. Meanwhile, we observed that hyaluronic acid (HA), heat shock protein 47 (HSP47), hydroxyproline (HYP), and other fibrosis indicators in both serum and liver tissues were significantly reduced in co-infection group [41]. Under normal circumstances, the levels of aspartate aminotransferase (AST) and alanine aminotransferase (ALT) in serum were relatively low. However, the membrane permeability increases when the corresponding cells are damaged, resulting in AST and ALT in cytoplasm releasing into the blood. In addition, the decrease of serum albumin (ALB) has important clinical significance to reflect the degree of liver function in fibrosis. ALB and AST levels increased in mono-infection group, but ALB levels were decreased. However, co-infection could partially restore the liver function. These results suggested that pre-infection with *T. gondii* could reduce liver fibrosis caused by schistosomiasis.

Liver fibrosis caused by schistosomiasis is closely coexists with T helper (Th) cell type 2-biased immunity, which usually related with imbalanced produce and degrade of collagen, or excessive extracellular matrix (ECM) deposition. Th1 cells can effectively inhibit the proliferation and activation of hepatic stellate cells by producing cytokines such as Interferon (INF)-γ, thus down-regulating the fibrosis of schistosomiasis. On the contrary, Th2 cells can produce a large number of cytokines like Interleukin (IL)-13 and transforming growth factor (TGF)-β, promoting the formation of fibroblasts and the deposition of collagen. Thus, a potential strategy against schistosomiasis-induced hepatic fibrosis is to increase Th1 response to coordinate Th1/Th2 balance in liver tissue [42,43]. In our study, we showed that TGF-β1 and IL-13 levels in both serum and liver tissue significantly increased after schistosomiasis infection, but decreased in the pre-infected *T. gondii* group, which indicated an inhibited Th2 response. Th1 and Th2 are a pair of mutual regulation cells. Co-infection may have a reciprocal regulatory effect on Th1 and Th2 responses. Whether Th cells differentiate into Th1 or Th2 on initial stage is interactively regulated by cytokines and other factors [6]. We showed that infection with schistosomiasis alone significantly increased the levels of IFN-γ and IL-4 in the host serum, while pre-infection with toxoplasma further improved the increasing effect of IFN-γ and soluble worm antigen (SWA)-IgG2a level but decreased the level of IL-4 and anti- soluble egg antigen (SEA)-IgG1. Our results were in accordance with the previous peer work, as they infused lentivirus carrying gra15II gene, which was an important effector molecules of *T. gondii*. Gra15II-activated liver macrophages were then percutaneously injected into a schistosomiasis mouse model, and found it played an inhibitory role in liver fibrosis [44]. Above all, our results revealed that the systemic Th2 response of co-infected mice were decreased, while the Th1 response was enhanced when compared with *S. japonicum* mono-infected mice. Therefore, we believed that chronic *T. gondii* prior infection with subsequent *S. japonicum* co-infection could reduce the level of liver fibrosis by inhibiting the activation of Th2 cells.

The gut microbiome (GM) plays a critical role in the pathogenesis of liver disease. With an imbalance of beneficial bacteria and pathobionts, or a hallmark event being dysbiosis, GM leads to a consequence of associated deleterious effects on the host. It has been reported that the diversity of intestinal microecology decreased in mice with acute *T. gondii* infection, which was mainly manifested by the growth of gram-negative bacteria and the transfer of symbiotic microecology to the liver. Further studies showed that acute toxoplasma infection could induce inflammatory bowel disease with expected microbiome changes in mice [45]. However, the long-term effect of *T. gondii* infection on the gut microflora are barely known. The inflammatory peak in gut is associated with *T. gondii* infection load, but this inflammatory response can reach a dynamic equilibrium in the host within five weeks after infection. What would happen to the intestinal microecology during *T. gondii* chronic infection has not been studied yet. On the other hand, peer works showed that *S. japonicum* infection modified bacterial richness in the fecal. GM communities were dominated by Helicobacter, Oscillibacter, Peptoclostridium, and Flavonifractor at the genus level, with a decreased amount of Lachnospiraceae_NK4A136_group [46] in *S. japonicum* chronic infected C57BL/6J mice (8 weeks p.i.). These data indicated a role of the mammalian liver-gut axis in the pathogenesis of schistosomiasis infection. However, to our knowledge, previous studies have not referred to the effect of *T. gondii* and *S. japonicum* co-infection on the GM community of the hosts. Above researches were partially in line with our observations, while the mice strain and *S. japonicum* infection scheme the colleagues used were different from ours. Both of us defined the relative abundance of Helicobacter (a subgroup of Epsilonbacteraeota at phylum level), Clostridium_sensu_stricto_1 (subgroups of Firmicutes at phylum level), and Escherichia_shigella (a subgroup of Proteobacteria at the phylum level) increased in *S. japonicum* mono-infected group. However, we firstly clarified the relative abundance of these taxa were further increased in co-infection cohort with a synergistic effect of *T. gondii*.

The microecology of liver diseases has become a research focus in recent years. Preclinical findings suggest that intestinal flora may be a driving factor for the development and progression of different liver diseases. Although, in our works, an overall reduction in GM alpha diversity in co-infected group was found compared with mono-infected samples, but the community of co-infected group were featured by expanded populations of dominant bacterial community. Among them Epsilonbacteraeota is reassigned as preponderant species in microbial communities as a novel phylum [47,48]. Peer works also showed that mice depleted with Escherichia-Shigella, secondary bile acid-producing bacteria, after antibiotic treatment, were resistant to metabolic improvement [49]. Bacteroides and Clostridium are butyrate-producing Flora [50,51] which were accompanied by synergistic augmentation with *T. gondii* pre-infection. Furthermore, butyrate has been shown to improve intestinal barrier integrity to maintain colonic health through promotes fatty acid oxidation [52,53]. In our studies, Clostridiaceae was identified as the biomarker with a significant difference between co-infected group and the other three groups at the family level, which is a subgroup of Clostridium_sensu_stricto_1. To date, details about Epulopiscium (subgroups of Firmicutes at phylum level) and their role in the gut of human hosts are lacking. However, it was worth mentioned here, Clostridium species were their closest relatives [54]. Interestingly, in our studies, the median of Epulopiscium in co-infected group was 5.3%, but its percentage in the rest groups was all 0%. The above results demonstrated that, although the diversity of intestinal flora in co-infection group decreased, but the abundance of preponderant flora significantly increased. Taxonomy results together with hierarchical cluster analysis and RDA/CCA all indicated that the liver fibrosis and Th1/Th2 immune response parameters could shape the taxonomy composition.

Our studies had some limitations. First, no attempts were made to measure the intestinal microbiome at more than one time point. Since most immune response has time-dependent manner during infection. It will be meaningful to assess gut microbiome alteration at additional time points throughout the whole process. Second, in this study, the effects of chronic *T. gondii* prior infection on *S. japonicum*, which induced Th2 differentiation and intestinal microecological changes were considered. While, further research should be conducted to determine the role of key bacteria in the progression of hepatic fibrosis. Finally, in addition to the direct effect of bacteria on the hosts, the regulation of metabolites produced by bacteria is also worth further discussed. Further studies on the correlation between microbiome changes and Th1/Th2 response related to chronic liver fibrosis, will be pursued.

Despite these limitations, this is the first study reported that prior *T. gondii* infection would lead to long-term alterations to the hosts’ GM, which further alleviated liver fibrosis induced by *S. japonicum* infection through drifting Th1/Th2 response. To the best of our knowledge, no reports have focused on the chronic effects of *T. gondii* prior infection (for eighteen weeks) on *S. japonicum* infected mice in vivo, as hepatic fibrosis had fully developed during that chronic episode. Most co-infection studies are concerned with the effects during acute phase (less than eight weeks). However, it’s worth mentioning that hosts are more susceptible to chronic superimposed effect of different parasites, instead of acute ones. Therefore, our modeling strategy was more in line with actual daily situations. Co-infections of parasites are often ignored in clinical, but this concomitant injury factor should always be considered.

## 4. Methods

### 4.1. Mice

All animal experimental protocols including *Toxoplasma gondii* (*T. gondii*), *Schistosoma japonicum* (*S. japonicum*) infection and anesthesia, were approved by the Animal Care and Use Committee at Jiangsu Institute of Parasitic Diseases (Ethics statement NO. IACUC-JlPD-2017010, 6 March 2017). The experimental procedures were carried out according to the Guide for the Care and Use of Laboratory Animals published by the US National Institutes of Health (NIH publication, 8th edition, 2011). Six-week-old female SPF ICR mice in our experiments were purchased from Comparative Medicine Center of Yangzhou University, China. The mice were housed in a humidity and temperature-controlled room on a light/dark cycle of 12 h, with free access to water and food.

### 4.2. Parasites and Infection of Mice

The cercariae of *S. japonicum* were obtained from 50 infected snails (*Oncomelania hupensis*, with sex ratio 1:1). The cysts of *T. gondii* were obtained from the brains of ICR mice 3 months after oral infection with cysts. For the *T. gondii* cohort, mice were orally infected with *T. gondii* cysts (strain Prugniaud type II) in 0.3 mL normal saline (including 6 cysts). Control group was inoculated with 0.3 mL normal saline by gavage (group: Ctrl). *T. gondii* group was oral infected with *T. gondii* cysts for totally 18 weeks (group: Tg). Abdominal adherent infection with 15 *S. japonicum* cercariae (strain Chinese Mainland) was conducted (group: Tg_Sj) after six weeks of *T. gondii* infection. Additional twelve weeks post infection of *S. japonicum*, mice were anesthetized and humanely sacrificed. *S. japonicum* infected group was conducted for totally 12 weeks as described above (group: Sj). Seven days after oral infection of *T. gondii*, whole blood from infected mice was collected for PCR detection of Toxoplasma B1 gene to verify the success of infection. The infection of *S. japonicum* was identified by the examination of parasite eggs from feces 4 weeks after abdominal the adherent infection of cercariae.

### 4.3. Histological Assessment

The specimens of liver tissues were fixed with 10% neutral formaldehyde solution and embedded in paraffin. After dewaxing, the sections were put into hematoxylin aqueous solution and stained. Thereafter the specimens were infiltrated into 70% ethanol and then into 90% ethanol dehydrated for 10 min. HE, Sirius red staining and Masson staining were carried out respectively. HE slides were dyed by eosin ethanol solution. Sirius red staining was carried out with Sirius red picric acid dye (1 g sirius red dissolved in 100 mL picric acid saturated solution). Masson dyeing was soaked by Masson lichun red acidic compound solution. Then, 1% phosphomolybdate aqueous solution was applied then and directly stained with aniline blue. Further, 0.2% glacial acetic acid solution was finally applied. The sections were sealed before observation after dehydration and transparency. The liver sections stained with HE, Masson and Sirius red stained sections were then assessed using a light microscope (Carl Zeiss, Axio Imager Z2, Germany).

### 4.4. Serum Factor Detection by ELISA

After infection, whole blood was collected from mice of each group. Serum supernatant was obtained after centrifugation at 10,000 r/min. The concentrations of TGF-β1, IL-13, IFN-γ and IL-4 in serum of mice were determined by ELISA Kit (Beyotime, Shanghai, China) according to the Manufacturer’s protocols. For soluble worm antigen (SWA) and soluble egg antigen (SEA) detection, SWA and SEA were coated with enzyme-labeled plates at the optimal concentration of 4 mg/L and sealed with 40 g/L skim milk in advance. After elution, anti-mouse IgG2a and anti-mouse IgG1 labeled with HRP were added to the plate labeled with SWA and SEA, respectively. TMB color rendering was performed after elution, the absorbance was immediately detected at the wavelength of 450 nm by Biotek microplate reader (Winooski, VT, USA).

### 4.5. Stimulation of Spleen Cells with SEA

Grind the spleen tissues with forceps, filtrate the cell suspension through 200 stainless steel screen mesh and centrifuge (1500 rpm) for 3 min. Aliqout the supernatant, crack the red blood cells with 2 mL lysis buffer for 3 to 5 min at room temperature and then centrifuge (1500 rpm) for 5 min. Fully suspend cell precipitation with 5 mL 1640 culture medium containing 10% fetal bovine serum. Adjust cell concentration to 5 × 10^6^/mL. Add 100 μL SEA (10 μg/mL) into 100 μL cell suspension and incubate the cell culture plate in 5% CO_2_, 37 °C incubator for 72 h. Centrifuge and collect the culture supernatant to detect the cytokine levels as described above.

### 4.6. Quantitative Real Time-PCR

Total RNA from liver tissues was extracted using Trizol reagent following the manufacturer’s instructions. Equal RNA was used to generate cDNA with HiScriptQ RT SuperMix (Vazyme, Nanjing, China). ChamQTM SYBR^®^ qPCR Master Mix (Vazyme, Nanjing, China) was used in real-time quantitative PCR. The average cycle thresholds (Ct) were calculated by using 2-ΔΔCT method which was used to calculate relative gene expression levels [55]. The primers for HSP47: 5′ -ACC GAG CCC TCT TCA GTC TT- 3′ (Forward), 5′-GGT GAT GCC CAA CAT AAC AAT- 3′(Reverse). The primers for TGF-β1: 5′ -GAA GTG GAT CCA CGA GCC CAA G- 3′(Forward), 5′- GCT GCA CTT GCA GGA GGG CAC -3′ (Reverse). The primers for type I collagen: 5′ -CGC CAT CAA GGT CCT ACT GC- 3′(Forward), 5′- ACG GGA ATC CAT CGG TCA -3′ (Reverse). The primers for type III collagen: 5′ -CCC AAC CCA GAG ATC CCA TT -3′(Forward), 5′- GAA GCA CAG GAG CAG GTG TAG A -3′ (Reverse). The primers for α-SMA: 5′ -GTC CCA GAC ATC AGG GAG TAA -3′(Forward), 5′- TCG GAT ACT TCA GCG TCA GGA -3′ (Reverse). The primers for IL-4: 5′ -CTC ATG GAG CTG CAG AGA CTC TT -3′(Forward), 5′- CAT TCA TGG TGC AGC TTA TCG A -3′ (Reverse). The primers for IFN-γ: 5′ -GGA TAT CTG GAG CTG GCA A -3′(Forward), 5′- TGA TGG CCT GAT TGT CTT TCA A -3′ (Reverse). The primers for IL-13: 5′ -CAC ACA AGA CCA GAC TCC CC -3′(Forward), 5′- CCA GGG ATG GTC TCT CCT CA -3′ (Reverse).

### 4.7. Sample Collection of 16s rRNA Sequencing

Specimen processing method with reference to the literature [1], for fecal pellets 1 g plus 9 mL PBS (0.05 mmol/L, pH7.4), sufficient oscillation and mixed for 5 to 10 min and low-speed centrifuge (500 r/min) for 5 min. Repeat high-speed centrifuge (9000 r/min) 3 min for 3 times. Aliqout the supernatant and crack the bacteria with 1% Triton X-100, thereafter heat the sample to 95 °C for 5 min.

### 4.8. DNA Extraction and Bacterial 16S rRNA Gene Sequencing

Briefly, microbial DNA was extracted from fecal pellets samples using the E.Z.N.a.^®^ soil DNA Kit (Omega Bio-tek, U.S.), The final DNA purification and concentration were determined by NanoDrop 2000 UV-vis spectrophotometer (Thermo Scientific, Wilmington, USA), and DNA quality was checked by 2% agarose gel electrophoresis. The V3-V4 hypervariable region of the bacteria 16S rRNA gene was amplified with primers F (5′-ACTCCTACGGGAGGCAGCAG-3′) and primer R (5′-GGACTACHVGGGTWTCTAAT-3′) by thermocycler PCR system (GeneAmp 9700, ABI, USA). PCR reactions were performed in triplicate 20 μL mixture containing 2 μL of 2.5 mM dNTPs, 4 μL of 5 × FastPfu Buffer, 0.4 μL of FastPfu Polymerase, 0.8 μL of each primer (5 μM) and 10 ng of template DNA. Urified amplicons were pooled in equimolar and paired-end sequenced (2 × 300) on an Illumina MiSeq platform (Illumina, San Diego, USA) according to the standard protocols by Majorbio Bio-Pharm Technology Co. Ltd. (Shanghai, China).

### 4.9. Bioinformatic Analysis

The orignial fastq files werefiltered by Trimmomatic and merged by FLASH according to the following standards: (i) Any site of which average quality score is less than 20 over a fifty base pairs (bp) sliding window, the reads will be truncated. (ii) The sequences with overlapping length greater than 10 bp are merged according to their overlap, which the mismatch should be less than 2 bp. (iii) The sequences of each sample were separated according to primers and barcodes (full match), reads could not begin with ambiguous bases and two mismatching nucleotides were allowed. Using UPARSE (version 7.1 http://drive5.com/uparse/) and a new “greedy” algorithm that performs both chimera filtering and OTU clustering, the two-stage sequences are read into operational taxonomic units (OTUs), and the clustering is carried out with 97% similarity cut-off value. RDP classifier algorithm (http://rdp.cme.msu.edu/) is used to analyze the classification of each 16S rRNA gene sequence. The confidence threshold of Silva (ssu123) 16S rRNA database is 70%. Redundancy analysis (RDA) was used to determine the relationship between environmental variables and bacterial community composition and visualize it using the R packages vegan. Using variance inflation factors “VIFs” to remove the VIFs > 10 in RDA model by “vif.cca” function.

### 4.10. Statistical Analysis

Comparisons within two groups were performed using one-way ANOVA analyzed with SPSS 18. 0. Differences with *P* value < 0.05 were regarded as significant. Statistical diagrams were made by GraphPad Prism 8. 0 software.

## 5. Conclusions

Above all, our data revealed that hosts with chronic prior *Toxoplasma gondii* (*T. gondii*) promoting immunosuppression to Th2 response caused by schistosoma infection. *T. gondii* prior infection promoted the expansion of preponderant flora in *S. japonicum*-infected mice, thus may led to a consequence of improved GM environment, which helped attenuate the progression of liver fibrosis through the gut-liver axis. Our studies may offer an increased understanding of the mechanisms by which *T. gondii* infection alters S. *japonicum*-induced liver fibrosis, and subsequently, hint towards improved strategies for the intervention of Th1/Th2 immune response or intestinal microecology in co-infected individuals.
